# O.4.4-1 Moving messages: development of the physical activity messaging framework and checklist

**DOI:** 10.1093/eurpub/ckad133.198

**Published:** 2023-09-11

**Authors:** Chloë Williamson, Paul Kelly, Graham Baker

**Affiliations:** Physical Activity for Health Research Centre (PAHRC), Institute for Sport, Physical Education and Health Sciences, University of Edinburgh, Edinburgh, UK; chloe.williamson@ed.ac.uk; Physical Activity for Health Research Centre (PAHRC), Institute for Sport, Physical Education and Health Sciences, University of Edinburgh, Edinburgh, UK; chloe.williamson@ed.ac.uk; Physical Activity for Health Research Centre (PAHRC), Institute for Sport, Physical Education and Health Sciences, University of Edinburgh, Edinburgh, UK; chloe.williamson@ed.ac.uk

## Abstract

**Purpose:**

Physical activity (PA) messaging is an important step in the pathway towards improving population PA levels, but best practice is not yet understood. A gap in the literature existed for a PA messaging framework to help guide creation and evaluation of messages. This study aimed to further develop and improve, and gain international expert consensus on, a Physical Activity Messaging Framework and Checklist.

**Methods:**

A modified Delphi study consisting of three online survey rounds was conducted. A preliminary version of the framework and checklist developed using findings from a scoping review of PA messaging were used as a starting point for this study. Each survey gathered feedback from an international expert panel using quantitative and qualitative methods. The framework and checklist were amended each round based on survey results until consensus (defined *a priori* as 80% agreement) was reached.

**Results:**

The final expert panel (n = 40, 55% female) came from nine countries and comprised academics (55%), healthcare and other professionals (22.5%) and government officials or policymakers (22.5%). Consensus was reached in survey 3 with 85% and 87.5% agreement on the framework and checklist, respectively. The Physical Activity Messaging Framework (PAMF) and Checklist (PAMC) comprise several PA messaging concepts organised into three overarching sections: (1) who, when, what, how and why, (2) message content, and (3) message format and delivery.

**Conclusion:**

This study presents an expert- and evidence-informed framework and checklist for physical activity messaging. The PAMF and PAMC can be used to create physical activity messages, plan evaluation of messages, and aid understanding and categorisation of existing messages. The PAMF and PAMC may improve practice by encouraging development of evidence-based and target audience-focused messages, as well as enhance the research base on physical activity messaging by harmonising key terminologies and improving quality of reporting.

**Funding source:**

This research was conducted as part of CW’s PhD, funded by the University of Edinburgh’s Principal’s Career Development Scholarship, Oct 2018 - Jan 2021.

